# The Origin and Evolution of RNase T2 Family and Gametophytic Self-incompatibility System in Plants

**DOI:** 10.1093/gbe/evac093

**Published:** 2022-06-17

**Authors:** Shouzheng Lv, Xin Qiao, Wei Zhang, Qionghou Li, Peng Wang, Shaoling Zhang, Juyou Wu

**Affiliations:** Centre of Pear Engineering Technology Research, State Key Laboratory of Crop Genetics and Germplasm Enhancement, Nanjing Agricultural University, Nanjing, China; Centre of Pear Engineering Technology Research, State Key Laboratory of Crop Genetics and Germplasm Enhancement, Nanjing Agricultural University, Nanjing, China; Centre of Pear Engineering Technology Research, State Key Laboratory of Crop Genetics and Germplasm Enhancement, Nanjing Agricultural University, Nanjing, China; Centre of Pear Engineering Technology Research, State Key Laboratory of Crop Genetics and Germplasm Enhancement, Nanjing Agricultural University, Nanjing, China; Centre of Pear Engineering Technology Research, State Key Laboratory of Crop Genetics and Germplasm Enhancement, Nanjing Agricultural University, Nanjing, China; Centre of Pear Engineering Technology Research, State Key Laboratory of Crop Genetics and Germplasm Enhancement, Nanjing Agricultural University, Nanjing, China; Centre of Pear Engineering Technology Research, State Key Laboratory of Crop Genetics and Germplasm Enhancement, Nanjing Agricultural University, Nanjing, China; Jiangsu Key Laboratory for Horticultural Crop Genetic Improvement, Nanjing, China

**Keywords:** RNase T2 family, gametophytic self-incompatibility, *S*-locus, phylogeny, evolution

## Abstract

Ribonuclease (RNase) T2 genes are found widely in both eukaryotes and prokaryotes, and genes from this family have been revealed to have various functions in plants. In particular, *S-RNase* is known to be the female determinant in the S-RNase-based gametophytic self-incompatibility (GSI) system. However, the origin and evolution of the RNase T2 gene family and GSI system are not well understood. In this study, 785 RNase T2 genes were identified in 81 sequenced plant genomes representing broad-scale diversity and divided into three subgroups (Class I, II, and III) based on phylogenetic and synteny network analysis. Class I was found to be of ancient origin and to emerge in green algae, Class II was shown to originate with the appearance of angiosperms, while Class III was discovered to be eudicot-specific. Each of the three major classes could be further classified into several subclasses of which some subclasses were found to be lineage-specific. Furthermore, duplication, deletion, or inactivation of the *S/S*-like-locus was revealed to be linked to repeated loss and gain of self-incompatibility in different species from distantly related plant families with GSI. Finally, the origin and evolutionary history of *S*-locus in Rosaceae species was unraveled with independent loss and gain of *S-RNase* occurred in different subfamilies of Rosaceae. Our findings provide insights into the origin and evolution of the RNase T2 family and the GSI system in plants.

**Significance**

RNase T2 genes participate in a variety of biological processes in plants, and some RNase T2 members play important roles in gametophytic self-incompatibility (GSI). However, the understanding of origin and evolution of RNase T2 family and GSI system in green plant remain limited. Herein, comprehensive identification and phylogenetic analysis of RNase T2 genes were performed in 81 sequenced plants. Evolutionary routes underlying transitions between self-incompatibility and self-compatibility in different plant species were demonstrated. The origin and evolution of *S*-locus in Rosaceae species were revealed to be related to independent loss and gain of *S-RNase*. The results of this study will enhance our understanding about the evolutionary history of RNase T2 family and the GSI system in plants.

## Introduction

Ribonucleases (RNases) are ubiquitous enzymes that participate in many cellular functions, including DNA replication, RNA catalysis, control of gene expression, and defense against microorganisms (MacIntosh 2011). The *RNases* are contained in a gene superfamily that includes different types of ribonucleases, which locate on membranous areas. This superfamily can be divided into three subfamilies: RNase A, RNase T1, and RNase T2 (Irie 1999). The RNase T2 family is found in almost all organisms, including animals, plants, viruses, and some bacteria. Hence, it is known to play important roles in eukaryotes and prokaryotes (Hillwig et al. 2009). Plant phylogenetic analysis has shown that the RNase T2 family underwent vast expansion, accompanied by gene duplication and loss (MacIntosh 2011). This family can be subdivided into three classes (Igic and Kohn 2001; Steinbachs and Holsinger 2002). Class I gene members are functionally diverse and commonly tissue-specific, Class II members are involved in biotic and abiotic stress responses, with some members that have housekeeping roles being highly conserved (MacIntosh et al. 2010; Hillwig et al. 2011; MacIntosh 2011). Class III members are exclusively found in core eudicots and comprise most of the *S-RNases* that are closely related to self-incompatibility (SI) in flowering plants (Ramanauskas and Igic 2017).

Recently, the RNase T2 family has been identified in many plant families, including Rosaceae (Vieira et al. 2021), Rutaceae (Liang et al. 2017), Solanaceae (Kothke and Kock 2011), and legumes (Azizkhani et al. 2021). Moreover, evolutionary analysis of the RNase T2 family has been conducted in *Arabidopsis thaliana, Nicotiana alata, Solanum lycopersicum, Pyrus persica*, and many other plants (Igic and Kohn 2001; MacIntosh et al. 2010; Morimoto et al. 2015; Ramanauskas and Igic 2017). However, the origin and evolutionary history of the RNase T2 family are largely unknown. Due to the advent of plant genome sequencing, genome-wide identification and phylogenetic analysis of the RNase T2 family have been conducted in various plants such as *A. thaliana*, tomato (*S. lycopersicum*), and rice (*Oryza sativa*) (MacIntosh et al. 2010; Hillwig et al. 2011; Kothke and Kock 2011; Megel et al. 2019). Previous studies have shown that Class I RNase T2 genes are contained in “early-diverging” groups (marchantiophyta, bryophytes, lycophytes, and ferns), some Class II RNase T2 genes are constitutively expressed (e.g., RNS2 in *A. thaliana* and RNase LER in *S. lycopersicum*) (Hillwig et al. 2011; Kothke and Kock 2011), and Class III genes may have originated from those in Class I or may share a common ancestor (MacIntosh et al. 2010). *S-RNases*, in Class III, have been the focus of much attention due to their important role in gametophytic self-incompatibility (GSI) (Ashkani and Rees 2016; Fujii et al. 2016; Zhao et al. 2021b). The molecular function of *S-RNase* genes has been studied in Rosaceae species such as *Prunus mume*, *Malus* × *domestica*, and *Pyrus bretschneideri*. Among these species, self-compatibility (SC) or SI is controlled by female-determinant *S-RNase* genes and male-determinant *S-locus F-box* (*SLF*) genes (Yaegaki et al. 2001; Wu et al. 2013; Pratas et al. 2018).

SI is widely found in flowering plant species, and diverse SI systems have evolved in different plant families (Fujii et al. 2016). Typically, SI systems can be classified into two types based on the genetic control of the pollen SI phenotype; these are GSI and sporophytic SI (SSI) (Zhang et al. 2009; Fujii et al. 2016). GSI is controlled by the *S*-locus, which contains the *S-RNase* and *F-box* genes; the pistil determinant of GSI is the *S-RNase* gene and the male determinants are *SLF* genes, which are usually linked to the *S-RNase* and are also known as *S-haplotype specific F-box* (*SFB*) genes (Sijacic et al. 2004). Recently, SI was divided into four specific types (Zhao et al. 2021b). Type-1 SI, found in most GSI species, is controlled by multiple pollen-specific *SLF*s from one haplotype and pistil-specific *S-RNases* from other haplotypes. Type-2 SI is found in the Brassicaceae and has been well studied; it is controlled by a male *S*-locus cysteine-rich protein/*S*-locus protein 11 and a female *S*-locus receptor kinase (Schopfer et al. 1999). Another GSI system, Type-3, is found in Papaveraceae, which possesses the common poppy stigma *S* (*PrsS*) and pollen *S* (*PrpS*) (Wheeler et al. 2009). Type 4 represents the sporophytic heterostyly of Primula, involving the *S*-locus supergene consisting of five style-encoding genes (Huu et al. 2016).

*S-RNase*-based GSI has been found in the common ancestor of Asteridae and Rosidae (Vieira et al. 2008), and *S-RNase* and *SLF* genes show co-evolution patterns in this system (Kubo et al. 2010). To date, this GSI system has been identified in five families (Solanaceae, Plantaginaceae, Rosaceae, Rutaceae, and Rubiaceae) (Franklin-Tong and Franklin 2003; McClure and Franklin-Tong 2006; Vieira et al. 2008, 2021; Zhao et al. 2021b). GSI occurs in two divergent patterns (in Maleae and Amygdaleae) in Rosaceae, in which different numbers of pollen-specific *SLF*s genes imply that there are different evolutionary mechanisms of self-pollen recognition in Maleae and Amygdaleae (Hua et al. 2008; Kubo et al. 2010; Fujii et al. 2016). Rosaceae species include many economically important fruit trees and ornamental plants and showed great diversity of SC/SI. Genomic characteristics and molecular function of *S*-locus (*S-RNases* and *SLFs*) in Rosaceae species have been well studied (Entani et al. 2003; Sassa et al. 2007; Wu et al. 2013; Abdallah et al. 2020; Du et al. 2021). However, the origin and evolution of *S*-locus in pear and Rosaceae remain largely unknown. In recent years, genomes of a number of Rosaceae species have been available, providing a valuable opportunity to study the evolutionary history of *S*-locus in Rosaceae species based on high-resolution genome comparison. In addition, it is unclear how species transition from the SI system to SC, or how the SI system was lost and reacquired during the evolution of Rosaceae.

A large number of plant genomes have been sequenced in recent years, and this has provided a valuable opportunity to resolve the evolutionary history of the RNase T2 gene family and the SI system on a broad scale. In this study, we conducted a comprehensive identification of RNase T2 gene family members in 81 sequenced plants. Phylogenetic analyses, synteny network analyses, and the identification of gene duplication events were performed to uncover the evolutionary history of the RNase T2 family. The repeated loss and gain of SI in different lineages were found to be related to the duplication, deletion, or inactivation of the *S*/*S*-like-locus in plant families with a GSI system. Moreover, the origin and evolution of the *S*-locus in Rosaceae was uncovered based on macro- and microsynteny analyses, nonsynonymous substitution rates (*K*_a_), and the synonymous substitution rates (*K*_s_) calculation. The loss and gain of *S-RNases* events were linked to the specific evolution of the *S*-locus in the Maleae of Rosaceae. This study provides insights into the origin and evolutionary history of the RNase T2 gene family and the frequent transitions between SI and SC in plants.

## Results

### Identification and Phylogenetic Analysis of RNase T2 Family Genes in Plants

**Fig. 1.**
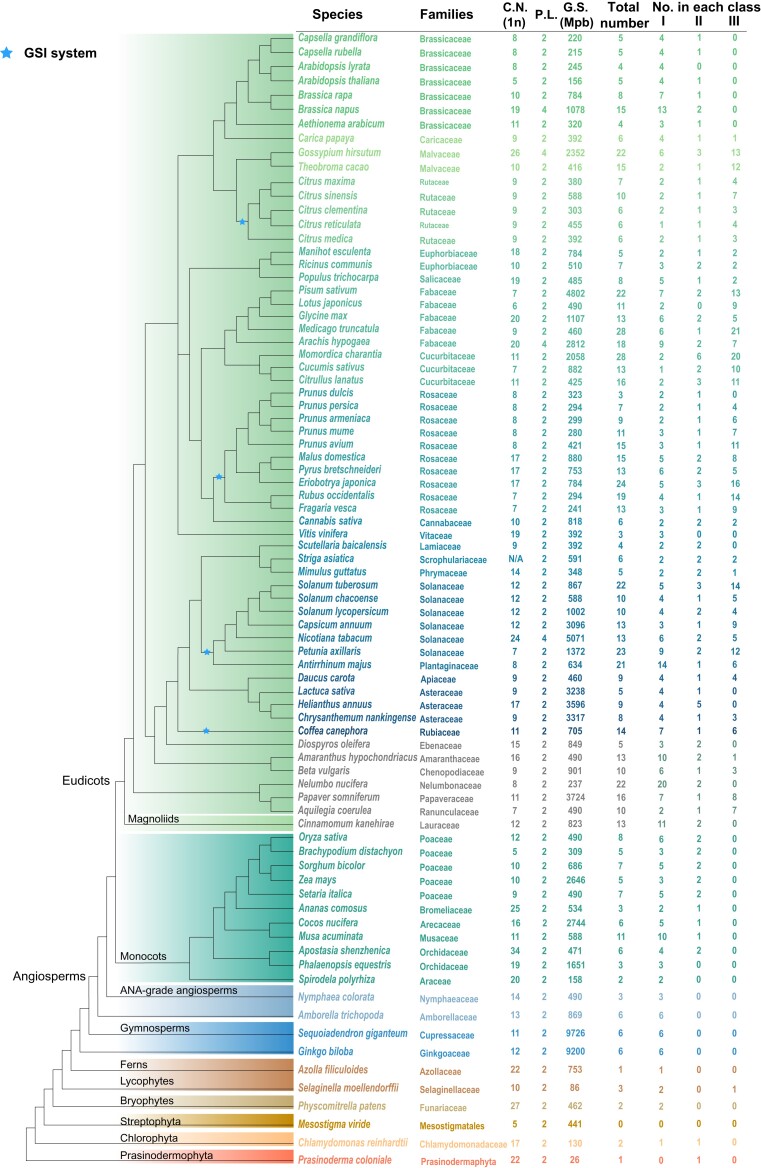
Phylogeny, genome information, and RNase T2 genes of 81 plant species. The taxonomic information and genome features of each species are presented. The total number of RNase T2 genes and the number in each class are shown for each species. C.N., haploid chromosome number (*n*); P.L., ploidy level; G.S., 1C genome size in megabase pairs (Mb). Information about genome size was obtained from the Plant DNA C-values Database (https://cvalues.science.kew.org/search).

HMMER3 was used to build the hidden Markov model (HMM) profile from the RNase T2 domain seed alignment file (PF00445) and to search against the whole-genome protein sequences of each species. In total, 785 RNase T2 genes were identified in 81 sequenced plant genomes covering diverse plant groups (supplementary table S1, Supplementary Material online). RNase T2 genes presented in almost all of the species investigated, ranging from green algae to angiosperms, suggesting their ancient origin. The number of RNase T2 genes varied greatly among different lineages and species (fig. 1). A higher number of RNase T2 genes was found in certain plant families, including Fabaceae (11–28 genes), Rosaceae (3–24 genes), Solanaceae (10–23 genes), Cucurbitaceae (13–28 genes), and Malvaceae (15–22 genes). The highest number of RNase T2 genes was found in *Medicago truncatula* and *Momordica charantia* (28 genes), while fewer were found in ferns, lycophytes, bryophytes, and green algae with the number of family genes ranging from 0 to 3.

**Fig. 2.**
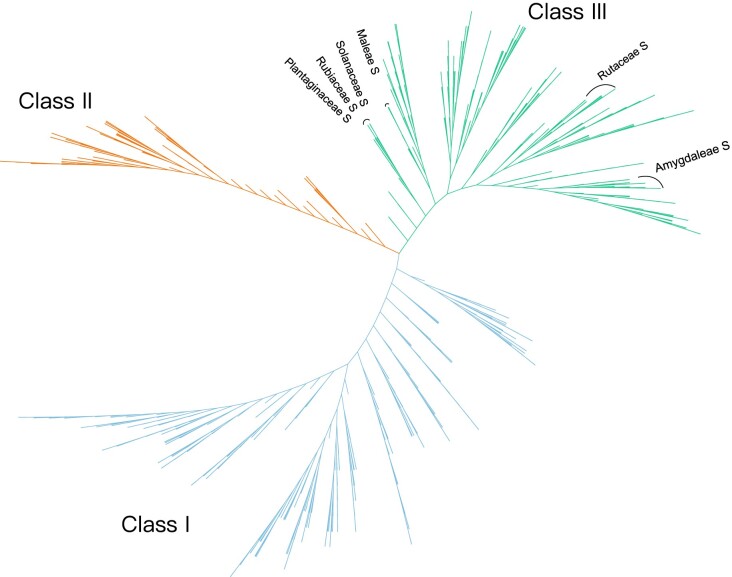
Phylogeny of RNases T2 family genes in 81 plants. The phylogenetic tree was constructed using IQ-TREE with the maximum-likelihood method and visualized using iTOL v6.3. The bootstrap was set to 1,000 replicates. Tip labels have been omitted for clarity. The RNase T2 genes were divided into three major classes (Class I, II, and III). *S-RNase* genes within Class III are indicated in five families with GSI, including the Rubiaceae, Plantaginaceae, Solanaceae, Rutaceae, and two genera of Rosaceae (Amygdaleae and Maleae). Support values of each branch are contained in supplementary figure S2, Supplementary Material online.

A phylogenetic tree of RNase T2 genes was constructed using IQ-TREE software with the maximum-likelihood method and a bootstrap value of 1,000. The RNase T2 family genes can be divided into three classes (I, II, and III) (fig. 2). In total, 353, 110, and 322 genes were classified into Class I, Class II, and Class III, respectively. A conserved motif analysis of the associated RNase T2 proteins was also performed, and the results supported the phylogenetic classifications (supplementary fig. S1, Supplementary Material online). The number of RNase T2 genes in each of the three classes was determined for each species (fig. 1). A large variation in the numbers of RNase T2 genes was observed among the three classes in a number of species. The number of RNase T2 genes in each class also showed great variation among different species (fig. 1). Class I members were detected in almost all investigated plants, suggesting the earlier origin of this class. The number of RNase T2 genes in Class I varied greatly among different species, but the number of Class I genes was generally higher than that of the other two classes. The high number of Class I genes was observed in some diploid species, including *Antirrhinum majus*, *Amaranthus hypochondriacus*, *Nelumbo nucifera*, *Cinnamomum kanehirae*, and *Musa acuminate,* as well as some polyploid plants, including *Brassica napus*, *Arachis hypogaea*, *Gossypium hirsutum*, and *Nicotiana tabacum*. Class II genes were more often found in monocot and eudicot plants and show no expansion, with a low number of genes (1–2) detected in the majority of species. Class III genes were specific to eudicot plants and included all known *S-RNase* genes involved in GSI, indicating that the S-RNase-based GSI system emerged following the diversification of eudicot plants. The expansion of Class III genes was observed in some families with GSI, including Malvaceae, Fabaceae, Solanaceae, Maleae, and Plantaginaceae. The absence of Class III genes was also observed in several eudicot plant families, including Brassicaceae, Asteraceae, Lamiaceae, and Vitaceae (fig. 1). The loss of Class III genes in Brassicaceae and Asteraceae species may be due to the specific type of SI (SSI system) that evolved in these two families.

To investigate the classification of each class of RNase T2, three phylogenetic trees were constructed using IQ-TREE software with the maximum-likelihood method and a bootstrap value of 1,000 (supplementary figs. S3–S5, Supplementary Material online). The results showed that Class I could be further divided into three subclasses (I-A, I-B. and I-C), and the support value of three subclasses was 62, 31, and 61 respectively (supplementary fig. S3, Supplementary Material online). Subclass I-A including 113 gene members was found to be specific to angiosperms, and Subclass I-B (108 members) was specific to dicots, while members in Subclass I-C (132) were detected from early-diverging green plants to eudicot plants, including species from prasinodermophyta, chlorophyta, streptophyta algae, bryophytes, lycophytes, ferns, gymnosperms, and angiosperms. Furthermore, Class II could be divided into three subclasses (II-A, II-B, and II-C) (supplementary fig. S4, Supplementary Material online), whose support values were 63, 35, and 41. Subclass II-A (50) was mainly composed of plants from Fabaceae, Rutaceae, and Brassicaceae. Members in Subclass II-B (22) are from monocots and several eudicot plant families like Lauraceae, Diospyros, Nelumbonaceae, and Caricaceae. The majority of Subclass II-C members (38) were from Solanaceae and Rosaceae. Class III could be divided into five subclasses (III-A, III-B, III-C, III-D, and III-E) with support values in 36, 68, 69, 50, and 68 (supplementary fig. S5, Supplementary Material online). Subclass III-A (49) was specific to Fabaceae, and members Subclass III-B (51) were only detected in Rosaceae species. Subclass III-C (65) members were from Rosaceae and Cucurbitaceae and Subclass III-D (77) incorporated genes from many eudicot plant families such as Plantaginaceae, Solanaceae, Rubiaceae. Subclass III-E (80) comprised genes from only three plant families including Solanaceae, Rosaceae, and Rutaceae. Interestingly, the *S-RNases* identified in Rosaceae species were only observed in Subclass III-C, and *S-RNases* in Plantaginaceae, Rubiaceae and Solanaceae only found in Subclass III-D, while *S-RNases* in Rutaceae were only observed in Subclass III-E. This result supported that the *S-RNases* have experienced lineage-specific evolution in different plant families during the speciation and diversification of core eudicots.

### Synteny Network Analysis of RNase T2 Family Genes

**Fig. 3.**
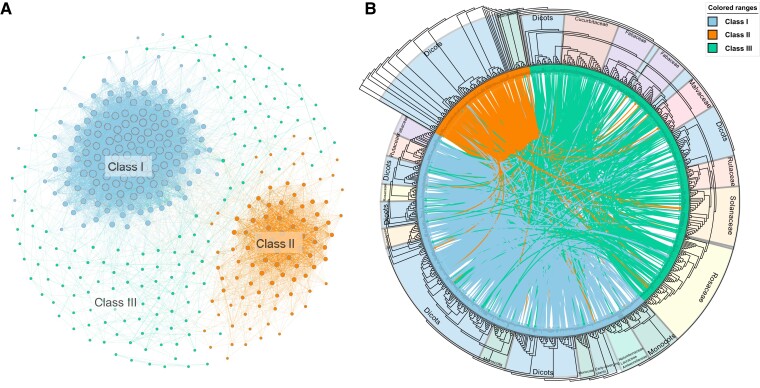
Synteny network of RNases T2 family genes and syntenic relationships within and between three classes of genes. (*A*) The synteny network of RNase T2 family genes. Communities were rendered based on the clique percolation method at *k* = 3. The size of each node indicates the number of connected edges (node degree). The communities are denoted by the three classes (Class I, II, and III) involved. (*B*) Syntenic relationships among the RNase T2 genes within the phylogenetic tree. Each connecting line located inside the inverted circular gene tree indicates a syntenic relationship between two RNase T2 genes. Lineage information was contained in the branch.

To explore the syntenic relationships and evolutionary history of RNase T2 family genes, we performed intra- and interspecies genome comparisons and collinearity analysis for 81 plants. A database was built to incorporate whole-genome syntenic gene pairs identified between any two species using MCScanX. The syntenic relationships of the RNase T2 genes among 81 plants were retrieved from the database, and a synteny network for RNase T2 genes was built. The syntenic RNase T2 gene network contained 580 nodes (i.e., genes that connected with other genes based on syntenic relationships), which were linked by 8,858 edges (i.e., pairwise syntenic connections) (supplementary table S1, Supplementary Material online). The syntenic RNase T2 gene network was further resolved and visualized using Gephi software. According to previous studies, a clique size of *k* = 3–6 was considered to approximate the true number of communities/clusters in clustering syntenic relationships (Palla et al. 2005; Xie et al. 2013). Hence, the syntenic gene clusters were identified in the RNase T2 gene network using the *k*-clique percolation clustering method implemented in Gephi with *k* = 3 (fig. 3*A* and supplementary table S1, Supplementary Material online). The node size indicated the number of syntenic connections for each node. Three major clusters or groups were determined based on the above synteny network analysis, corresponding to the phylogenetic classification. The node size within Class I was larger than that for the other two classes, indicating that members of Class I have strong collinear relationships. Genes in the Class II group showed weaker synteny compared with those in Class I. Moreover, members of Class III had weak syntenic relationships and were scattered within the network, implying that Class III gene members may have evolved in lineage-specific ways, which resulted in nonconserved chromosome localizations. The syntenic RNase T2 gene network did not include members from *Physcomitrella patens*, *Mesostigma viride*, *Chlamydomonas reinhardtii*, or *Prasinoderma coloniale*, probably due to the rare occurrence of RNase T2 genes in these early-diverging green plants and the long-term evolutionary divergence from other species.

All of the homologous relationships (or gene pairs) within the syntenic RNase T2 gene network were linked and displayed in the phylogenetic tree of the RNase T2 genes in the 81 investigated plants (fig. 3*B*). The link colors used in the tree correspond to the colors of the different clusters (or classes) determined in the synteny network. The syntenic relationships among the RNase T2 genes within each class were shown to be strong, supporting the classification results from the phylogenetic analysis. In addition, a large number of syntenic gene pairs among the three classes were observed.

### Gene Duplication Events and Expansion of the RNase T2 Gene Family

**Fig. 4.**
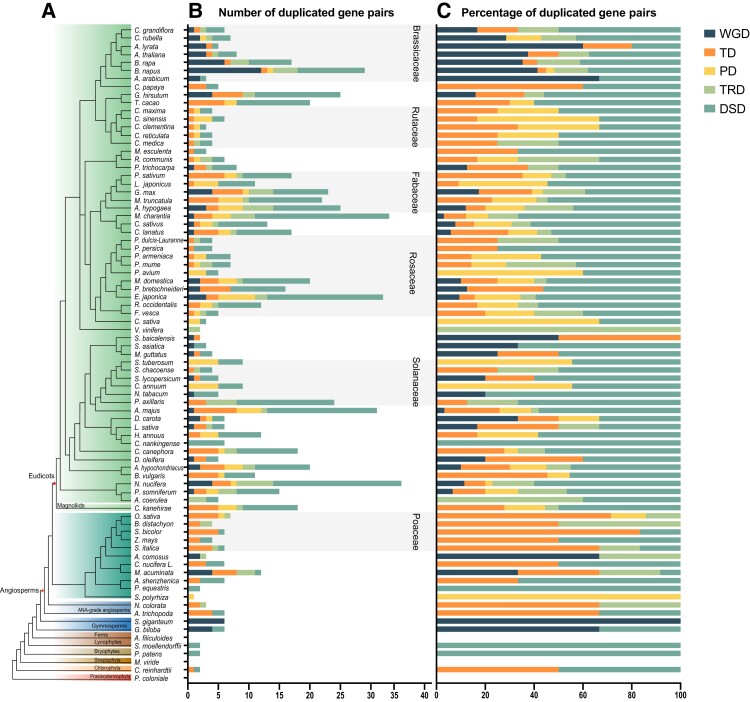
The number and percentage of RNase T2 gene pairs derived from different modes of gene duplication in 81 plant species. (*A*) Taxonomy tree of 81 plant species. (*B*) Number of duplicated gene pairs. Five modes of duplicated RNase T2 gene pairs were identified, and the different colored bars represent the different modes of gene duplication. WGD, whole-genome duplication; TD, tandem duplication; PD, proximal duplication; TRD, transposed duplication; DSD, dispersed duplication. (*C*) Percentage of duplicated gene pairs.

Gene duplication events in the RNase T2 family genes in 81 species were identified using the DupGen_finder pipeline (fig. 4 and supplementary table S2, Supplementary Material online). Five types of gene duplication events were detected, including whole-genome duplication (WGD), tandem duplication (TD), proximal duplication (PD), transposed duplication (TRD), and dispersed duplication (DSD). In those families or subfamilies that had not been influenced by recent WGDs, such as Rutaceae, Amygdaleae, Rosoideae, and Poaceae, single-gene duplication was found to contribute to RNase T2 gene family expansion. In other families, such as Brassicaceae, Fabaceae, Maleae, Solanaceae, and Plantaginaceae, which underwent more recent and lineage-specific WGD or whole-genome triplication events, genome duplication was the major force driving the expansion of the RNase T2 gene family. In addition, the expansion of the RNase T2 family was found in certain individual species that had experienced additional species-specific WGD, including *G. hirsutum* (Wang et al. 2012), *Mimulus guttatus* (Mühlhausen and Kollmar 2013), *Daucus carota* (D’hont et al. 2012), and *Musa acuminata* (Jiao et al. 2014). In the Gymnosperms, WGD was the major force driving expansion. However, in some early-diverging plants, such as *Azolla filiculoides*, *M. viride*, and *Pr. coloniale*, duplicated gene pairs were not identified.

### Evolutionary Transition of the S-RNase-Based Gametophytic Self-Incompatibility System in Plants

**Fig. 5.**
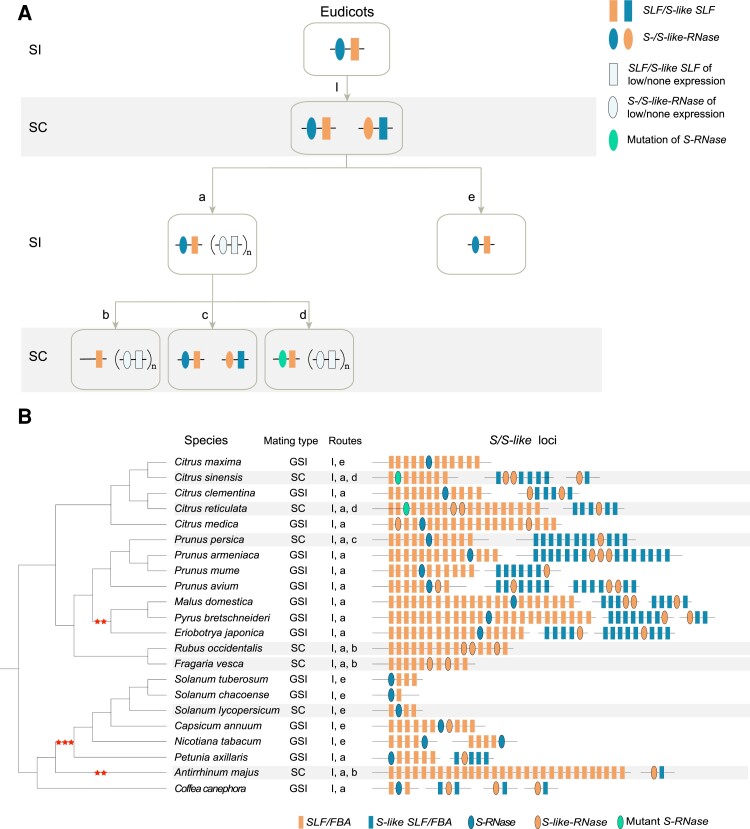
The *S*-/*S*-like loci annotated in species from five plant families with GSI and the evolutionary routes for the loss and regain of SI. (*A*) The evolutionary routes proposed to illustrate the loss and regain of SI. The loss of SI may be caused by duplication (I), deletion (b), reactivation (c), or mutation (d), while the regain of SI may be due to inactivation (a) or deletion of the *S*-like-locus (e). The solid-color rectangles and ovals represent *SLF/S-like SLF* and *S-/S-like-RNase* genes involved in GSI. (*B*) Annotated S-/S-like loci composed of *S-/S-like-RNases* and *F-box/FBA* genes in 22 angiosperm species from five plant families with GSI systems. The species with SC are highlighted with a gray background. Two and three stars indicate WGD and whole-genome triplication, respectively. The “Routes” (numbers and letters) correspond to the evolutionary processes depicted in (*A*). This figure was inspired by a previous study (Zhao et al. 2021b).

We annotated and analyzed the complete gametophytic *S*/*S*-like-locus in 22 species representing five eudicot families with the GSI system to explore the evolutionary transitions that occurred between SI and SC (fig. 5*A* and *B* and supplementary table S3, Supplementary Material online). Previous studies have indicated that the GSI system emerged in the common ancestor of eudicots, and repeated losses of the SI system have occurred during subsequent evolutionary events and through diversity, due to genome or segmental duplications, which resulted in the appearance of SC. WGDs or segmental duplications could generate a copy of the *S*-locus, resulting in competitive interaction between the two copies and the loss of SI through Route I (fig. 5*A*) (Zhao et al. 2021b). However, the majority of extant species exhibited SI, which led us to query how SI was regained in these species. Reduced expression or inactivation of *S-RNases* and *SLFs* in the duplicated copy of the *S*-locus have been suggested to lead to the regain of SI in *Antirrhinum hispanicum* (Fujii et al. 2016; Zhao et al. 2021b). Based on this observation, we inferred that some species in Rosaceae (*Prunus armeniaca*, *Pr. mume*, *Prunus avium*, *M. domestica*, *Py. bretschneideri*, and *Eriobotrya japonica*), Rutaceae (*Citrus maxima*, *Citrus clementina*, and *Citrus medica*), Solanaceae (*Petunia axillaris*), and Rubiaceae (*Coffea canephora*) regained SI through reduced expression of the duplicated copy (*S*-like-locus) of the *S*-locus (Route a). Another route to regaining SI was thought to be the deletion of the *S*-like-locus (Route e). For instance, some species in the Solanaceae (e.g., *Solanum tuberosum, Solanum chacoense, N. tabacum*, and *Capsicum annuum*) contain a single *S*-locus with no counterpart copy. However, one species (tomato, *S. lycopersicum*) in the Solanaceae was shown to lose SI once again during domestication from wild tomato, which was attributed to the inactivation of *S-RNase*, although the *S*-locus still exists (Route e) (Tomato Genome 2012; Zhao et al. 2021b). The loss of SI could also occur via three further routes: deletion of *S-RNase* (Route b), reactivation of the *S*-like-locus (Route c), and mutation of *S-RNase* (Route d). In *A. majus* (Plantaginaceae), *S-RNase* at the *S*-locus was found to be lost after experiencing long-term artificial selection, leading to the transition from SI to SC (Route b) (Zhao et al. 2021b). Likewise, the loss of SI in some Rosaceae species (*Fragaria vesca* and *Rubus occidentalis*) may have occurred through deletion of *S-RNase* (Route b) (Du et al. 2021). In particular, one species (*Py. persica*) in Rosaceae lost SI, following the reactivation the *S*-like-locus (Route c). The loss of SI in two species (*Citrus reticulata* and *Citrus sinensis*) in the Rutaceae has been suggested to result from the mutation of *S-RNase* at the *S*-locus (Route d) (Liang et al. 2020), which *S-RNase* was truncated and its predicted protein lacks the C4 and C5 conserved domains, the HV4 and HV5 hypervariable domains and four conserved cysteine residues.

In addition, multiple sequence alignment of *S-RNases* from 22 species with GSI was performed to detect variations in the amino acid sequences and functional domains and their associations with evolutionary transitions between SI and SC (supplementary fig. S6, Supplementary Material online). Previous studies have demonstrated that conserved domains 1 and 2 represent the functional domains of RNase T2 genes and that histidine is the acting residue (MacIntosh et al. 2010). Here, we found that five conserved domains and the hypervariable region were preserved in the majority of *S-RNases*, suggesting high levels of sequence conservation in S-RNases. In particular, the *S-RNases* in *Ci. reticulata* and *Ci. sinensis* have shorter sequence lengths compared with *S-RNases* in other species, as they lack one or two conserved domains, hypervariable domains, and other conserved cysteine residues, which may be related to the loss of SI in these two species (Liang et al. 2020).

### The Origin and Evolutionary History of the *S*-locus in the Rosaceae

Diversified types of SI or SC are found in Rosaceae species. The *S*-locus, composed of *S-RNases* and *SLF* genes, is the key region for determining SI or SC. However, the origin and evolutionary history of the *S*-locus in Rosaceae have not been well studied. Therefore, we performed macro- and microsynteny analyses within and between genomes of seven Rosaceae fruit species (pear, apple, loquat, Japanese apricot, peach, bowman’s root, and apricot) and outgroup species (grape), among which pear (*P. bretschneideri*), apple (*M. domestica*), loquat (*E. japonica*), and Japanese apricot (*Pr. mume*) and apricot (*P. armeniaca*) are self-incompatible, while peach (*Py. persica*), bowman’s root (*Gillenia trifoliata*), and grape (*Vitis vinifera*) are self-compatible. Strawberry (*F. vesca*) and black raspberry (*R. occidentalis*) were used as outgroups for Amygdaleae and Maleae, because their genomes have not been influenced by recent WGD events, they have experienced fewer genome rearrangements, and they have preserved the chromosome structure of Rosaceae ancestors, moreover, they are both self-compatible. Grape was used as the outgroup for Rosaceae species which was also self-compatible. The ancestor of Maleae species underwent a recent and lineage-specific genome duplication event. However, only one *S*-locus was retained in extant Maleae species, raising the question as to how another duplicated *S*-locus was lost.

**Fig. 6.**
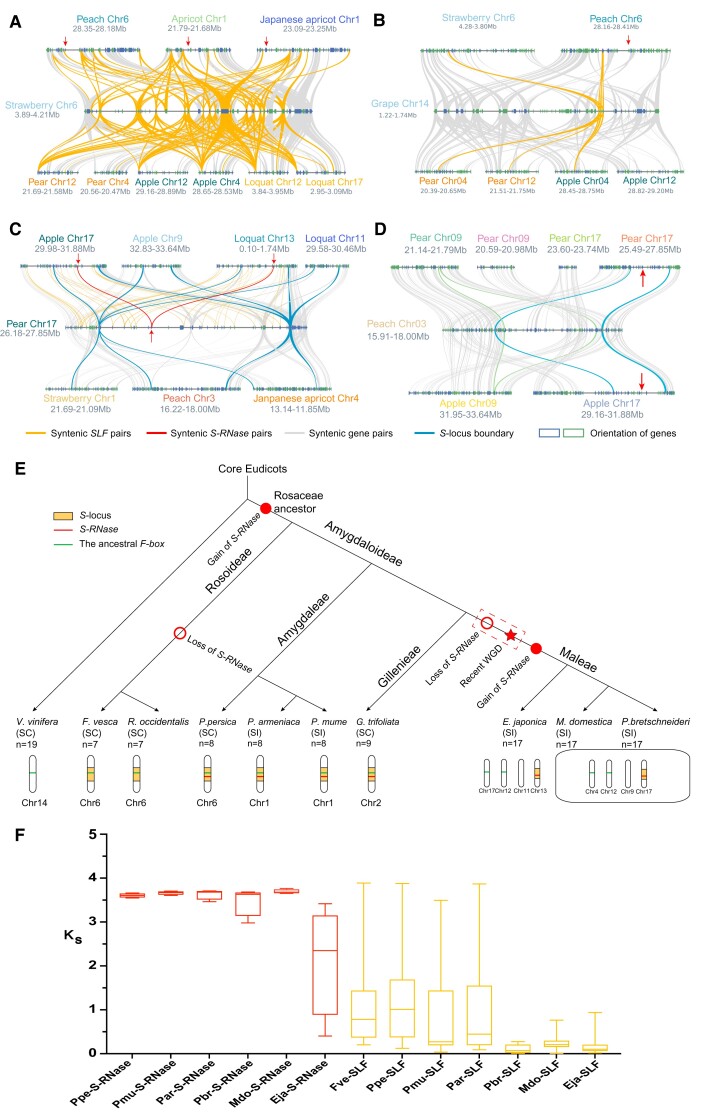
The origin and evolutionary history of the *S*-locus in Rosaceae. (*A*) Microsynteny relationships of the *S*-locus region in strawberry and homologous segments in Amygdaleae and Maleae species. The arrows indicate *S-RNases*. (*B*) Microsyntenic relationships between the *S*-locus region in strawberry and homologous segments in other species from Amygdaleae, Maleae, and Vitaceae. (*C*) Microsyntenic relationships between the *S*-locus region in pear and syntenic regions in other species of Rosaceae. (*D*) Microsyntenic relationships between the extended segment on peach Chr 3 and syntenic regions in pear and apple. (*E*) The evolutionary model proposed to illustrate the origin and diversified evolution of the *S*-locus in Rosaceae. (*F*) Boxplot of *K*_S_ distributions between *S*-locus genes (*S-RNases* and *SLFs*) and its closest paralog calculated in Rosaceae species.

Based on well-studied *S-RNases* and *SLFs* in Rosaceae fruit species (Entani et al. 2003; Sassa et al. 2007; Wu et al. 2013; Abdallah et al. 2020; Du et al. 2021), we can retrieve the location of *S*-locus regions in strawberry, peach, Japanese apricot, pear, and apple. To start, we performed pairwise macrosynteny analysis among strawberry and other Rosaceae fruit species (supplementary figs. S7*A* and S8, Supplementary Material online), and found that the *S*-locus on Chr 6 in strawberry has syntenic relationships with Chr 6 in peach, Chr1 in Japanese apricot and apricot, Chr 4 and 12 in pear and apple, and Chr 12 and 17 in loquat. However, there was no detectable synteny between the chromosomes containing *S-RNases* (Chr 17 in pear and apple and Chr 13 in loquat) in Maleae and those (Chr 6 in peach and Chr 1 in Japanese apricot and apricot) in Amygdaleae (supplementary fig. S7*B* and *C*, Supplementary Material online). Intergenomic synteny analysis between strawberry and other Rosaceae fruit species was performed to identify the syntenic regions of strawberry *S*-locus. Only one syntenic region was identified in Amygdaleae species (peach, apricot, and Japanese apricot) for strawberry *S*-locus (fig. 6*A*, supplementary table S4, Supplementary Material online). Interestingly, the chromosomal locations of syntenic regions identified in peach, apricot, and Japanese apricot are consistent with the locations of *S*-locus reported in previous studies (Sassa et al. 2007; Abdallah et al. 2020); however, no gene corresponding to the *S-RNases* in Amygdaleae was found in strawberry, indicating that *S-RNase* has been lost in strawberry. In addition, two syntenic regions could be found in each of Maleae species (pear, apple and loquat) for strawberry *S*-locus, while the syntenic regions (e.g., pear Chr4 and Chr12) identified in Maleae species are not the *S*-locus although several *F-box* genes were found to be syntenic with *SLFs* contained in strawberry *S*-locus. Therefore, it could be inferred that the ancestral *S*-locus with primitive *SLF* has appeared before the diversification of Rosaceae species, similar results were obtained when using black raspberry as the outgroup to perform the microsynteny analysis (supplementary fig. S9*A* and *B* and table S5, Supplementary Material online). However, the timing of origination of *S-RNases* is still undetermined, which were given to the following two possible hypotheses: (1) *S-RNases* emerged before the diversification of Rosaceae species, and subsequent gene loss of *S-RNases* occurred in Rosoideae species (e.g., strawberry Chr6) and Maleae species (e.g., pear Chr4 and Chr12), (2) *S-RNases* have not appeared in the common ancestor of Rosaceae until the divergence of Amygdaleae, that is, the independent origination of *S-RNases* occurred in the common ancestor of Amygdaleae species (peach, apricot, Japanese apricot).

To validate the above two hypotheses, we firstly performed intergenomic synteny analysis between strawberry and grape (outgroup) (fig. 6*B*). The result showed that one *SLF* gene (FvH4_6g06890) in strawberry *S*-locus region has conversed synteny relationships with an *F-box* gene within syntenic block identified in grape, and with *SLFs* in other Rosaceae species, also, syntenic gene or orthologous gene for peach *S-RNase* (Chr6) has not emerged in grape. This result supported the ancestral origin of *SLF* in the *S*-locus and provided evidence for the more recent origin of *S-RNase* after the split of grape and Rosaceae. Furthermore, we calculated the nonsynonymous substitution rates (*K*_a_) and the synonymous substitution rates (*K*_s_) between *S-RNase* or *SLF* and their putative homologous gene pairs in investigated Rosaceae species (fig. 6*F* and supplementary table S13, Supplementary Material online). Interestingly, we found the *K*_s_ value (∼3.9) between the aforementioned ancestral *SLF* (FvH4_6g06890) and its homologous gene (FvH4_1g04390) is higher than other homologous *SLF* gene pairs (supplementary table S13, Supplementary Material online). This result further supported that the ancestor of the *SLF* gene has emerged before the diversification of Rosaceae. In addition to this ancient *SLF* gene pair found in strawberry, other *SLF* gene pairs have lower *K*_s_ values (0.2–1.6) (supplementary table S13, Supplementary Material online), and the similar *K*_s_ values were found in other Rosoideae species (black raspberry), Amygdaleae species (peach, apricot, and Japanese apricot), and Gillenieae (bowman’s root). However, for Maleae species (pear, apple and loquat), the *K*_s_ values between *SLF* genes within *S*-locus and their homologous genes are very low (0.008–0.27), suggesting their more recent origin. If *S-RNase* occurred independently in the most recent common ancestor of Amygdaleae and Gillenieae, we anticipated a lower *K*_s_ value between homologous *S-RNase* gene pair in peach, apricot, Japanese apricot, and bowman’s root. However, the *K*_s_ values between *S-RNases* and their putative homologous genes are high (∼3.6), but slightly lower than the *K*_s_ values of the aforementioned ancient *SLF* gene pair (∼3.9) (supplementary table S13, Supplementary Material online). Therefore, these results supported “hypothesis 1” that the ancestral *SLF* has originated before the split of grape and Rosaceae ancestor, while the ancestral *S-RNase* emerged after the split of grape and Rosaceae ancestor and had an independent origin in the common ancestor of Rosaceae. Subsequent gene loss of *S-RNases* happened in Rosoideae species (e.g., strawberry Chr6) and Maleae species (e.g., pear Chr4 or Chr12), respectively. It is still unclear whether the loss of *S-RNase* occurred before WGD or not in Maleae, probabilistically speaking, the loss of *S-RNase* would happen before a recent WGD, because *S-RNase* need to be lost only once before WGD while need to be lost twice after WGD.

The *S*-locus in extant Maleae species has been found to be located in Chr17 (pear and apple) or Chr13 (loquat) (Sassa et al. 2007; Wu et al. 2013). We performed intergenomic synteny analysis between pear and other Rosaceae fruit species including strawberry (Rosoideae), peach (Amygdaleae), Japanese apricot (Amygdaleae), loquat (Maleae), and apple (Maleae). Pear *S*-locus has conserved synteny relationship with those in apple and loquat, suggesting that ancestral *S*-locus has emerged in the common ancestor of Maleae (fig. 6*C*). Meanwhile, we found that pear *S*-locus and surrounding region have syntenic relationships with blocks in strawberry (Chr1), peach (Chr3), and Japanese apricot (Chr4), respectively. However, no *SLF/SLF-like* genes or *S/S-like-RNases* were found in these blocks of strawberry, peach, and Japanese apricot, implying that they are not *S/S*-like-locus (supplementary table S8, Supplementary Material online). We further performed microsynteny analysis between peach and pear or apple using the block found in peach Chr3 as reference and found that this block has two syntenic regions in pear and apple (Chr9 and Chr17) (fig. 6*D*). The two blocks in pear Chr9 and Chr17 are homologous and derived from Maleae-specific WGD event but no *SLF/SLF-like* genes or *S/S-like-RNases* were found in the block in Chr9, suggesting more recent origin of *S*-locus in pear Chr17 after WGD. In addition, no synteny was found between the *S*-locus on pear Chr17 and *S*-locus on peach Chr6 or strawberry Chr6 (fig. 6*A* and *C* and supplementary fig. S7*D* and *E*, supplementary tables S7 and S8, Supplementary Material online), supporting the independent origin of *S*-locus in the common ancestor of Maleae after divergence with Rosoideae and Amygdaleae. Similar results were obtained from a microsynteny analysis between Japanese apricot (bowman’s root) and pear and apple (supplementary fig. S10*A*–*C* and tables S11 and S12, Supplementary Material online).

The common ancestor of Maleae underwent a lineage-specific WGD, if the gain of *S-RNase* and formation of *S*-locus occurred before the WGD event, we expected to find two copies of *S*-locus in Chr 9 and Chr 17, respectively, in the pear and apple genome. However, only one *S*-locus was observed in pear (or apple) by intragenomic synteny analysis and no *F-box* gene and T2 *RNases* were found in the block on Chr 9 (fig. 6*D*, supplementary table S9, Supplementary Material online), supporting that gain of *S-RNase* occurred after recent WGD.

Based on the results described above, we proposed an evolutionary model to illustrate the origin and evolution of the *S*-locus in Rosaceae (fig. 6*E*). The *S-RNases* were gained in Rosaceae ancestors and then generated *S*-locus together with ancestral *F-box* genes. Rosoideae species (strawberry and black raspberry) inherited the *S*-locus on Chr 6 but lost the *S-RNases*. Amygdaleae species (peach, Japanese apricot, and apricot) and Gillenieae species (bowman’s root) also inherited the *S*-locus and retained the *S-RNases*. The *S*-locus of peach was shown to be located on Chr 6, which is similar to that observed in rose (*Rosa chinensis*) (Hibrand Saint-Oyant et al. 2018), and the *S*-locus in Japanese apricot and apricot was found to be located on Chr 1. In Maleae, the *S-RNase* has undergone the loss event and has regained after a recent WGD event, resulting in the current nonconserved chromosomal location of the *S*-locus.

## Discussion

### The Ancient Origin and Lineage-Specific Expansion of the RNase T2 Family in Plants

The RNase T2 family comprises a type of endoribonuclease this is found ubiquitously in two domains of life (Eukarya and Bacteria) that include viruses, bacteria, protozoans, animals, and plants (Irie 1999; Luhtala and Parker 2010). The number of RNase T2 genes varies greatly among different species (Luhtala and Parker 2010). In this study, a total of 785 RNase T2 genes were identified from 81 sequenced plants representing diversified plant lineages. The size of the RNase T2 family was found to differ greatly among different species, with rare RNase T2 genes found in early-diverging plants such as green algae, moss, and fern, which is consistent with previous research (Ramanauskas and Igic 2017). The expansion of RNase T2 genes occurred in some plant families within eudicots such as Brassicaceae, Malvaceae, Rosaceae, Solanaceae, and Fabaceae, which can be attributed to lineage-specific genome duplications or single-gene duplication events. Single-gene duplications, especially PD, TD, and TRD, have contributed greatly to the expansion of the RNase T2 gene family in some families or genera, including Rutaceae, Amygdaleae from Rosaceae, and Poaceae. Based on a phylogenetic analysis, the RNase T2 gene family was divided into three subgroups (Class I, II, and III), and the phylogenetic classification was supported by an analysis of conserved motifs (Zhao et al. 2017). Moreover, the phylogeny of RNase T2 family genes was verified by synteny network analysis (Zhao and Schranz 2017), which was previously applied to infer the phylogeny of MADS-Box genes (Zhao et al. 2017), the TMBIM superfamily (Gamboa-Tuz et al. 2018), and fibrillarin (FIB) (Pereira-Santana et al. 2020). The number of RNase T2 genes was determined in each of the three classes of the 81 plant species. Class I genes presented in broad-scale lineages spanning from early-diverging green algae to angiosperms, suggesting the ancient origin of Class I genes. Class II genes were mainly found in angiosperms, while Class III genes containing *S-RNases* were only discovered in core eudicots (Hillwig et al. 2011), implying the more recent origin of this class. However, some exceptions were observed in this study. For example, the Class II genes were recognized to be angiosperm-specific; although one Class II gene was identified in the green algae species *C. reinhardtii* and *Pr. coloniale*, respectively. In addition, one Class III gene (eudicot-specific) was found in the lycophyte *Selaginella moellendorffii*. These exceptions may be due to the long-branch attraction when constructing the phylogenetic tree (Felsenstein 1978). The RNase T2 genes from the three classes have evolved to possess diversified functions. The members of Class I showed tissue-specific expression and were found to play important roles in abiotic and biotic stress (MacIntosh et al. 2010). The number of genes in Class II was lower than that in the other classes, with one or two members identified in the majority of plants. Moreover, some Class II members have been regarded as housekeeping genes (MacIntosh et al. 2010; Kothke and Kock 2011). The number of genes in Class III was higher in some plant families with a GSI system, which may be attributed to the high number of *S-like-RNases* (Hua et al. 2008). No Class III members were found in Brassicaceae species, implying that lineage-specific gene loss may have occurred in the ancestor of Brassicaceae. What is more, by using large-scale species information, we found more classification about subclasses of each class in RNase T2 family which never reported before. Class I, II, and III were first classified into 3, 3, and 5 subclasses, respectively. The results suggested that the evolution of each class members especially *S-RNases* were lineage-specific. Besides, *S-RNases* were distributed into three different subclasses which indicated that *S-RNases* probably originate independently in each plant families. The higher-resolution phylogenetic trees for each of three major classes of RNase T2 gene family have provided more information about the classification of RNase T2 family genes and enhanced our understanding about the evolution of RNase T2 genes. Based on syntenic network analysis, a high density of syntenic relationships was found between Class I/II and Class III, indicating that Class III genes may originate from Class I/II genes. Furthermore, the homologous counterparts of some *S-RNases* included in Class III were found in Class I/II, suggesting that *S-RNases* may be derived from Class I/II. Interestingly, a recent study found that the Class I/II T2 RNases and FBA/FBK genes were linked in monocots and basal angiosperms, and these links were suggested to represent the ancestral prototype of the *S*-locus found in extant eudicots (MacIntosh 2011; Zhao et al. 2021b).

### The Repeated Loss and Gain of SI

The S-/S-like loci were annotated and compared among species in plant families exhibiting GSI, and the evolutionary routes underpinning repeated loss and regain of SI were inferred and generalized according to the evolutionary model proposed recently to explain repeated transitions between SC and SI (Zhao et al. 2021b). Genome or segment duplication could generate a duplicated copy of the *S*-locus within the same genome, which would cause competitive interaction between two copies of *S-RNase* in pollen and lead to a loss of SI (Route I). The regain of SI can occur through inactivation or deletion of one copy of the *S*-locus. A previous study showed that one species (*A. hispanicum*) in Plantaginaceae contained a duplicated copy of the *S*-locus, in which the *SLF* and *S-like-RNase* presented low levels of expression in stamens and styles, resulting the regain of SI in this species. This suggested that SI could be regained via inactivation of duplicated copies (Zhao et al. 2021b). In addition, deletion, mutation, and inactivation of *S-RNases* have been suggested to represent three common routes that account for the repeated loss of SI (Zhao et al. 2021b). The deletion of *S-RNases* was found in some species of Rosaceae and Plantaginaceae exhibiting SC, such as *F. vesca*, *R. occidentalis* (Bošković et al. 2010; Aguiar et al. 2015; Du et al. 2021), and *A. majus* (Zhao et al. 2021b). The mutation of *S-RNase* has been found to be related to the transition from SI to SC. For example, the loss of SI in “Guiyou” pummelo (*C. maxima*, Rutaceae) was attributed to a pistil-side *Sm-RNase* mutation (Liang et al. 2020; Hu et al. 2021). Moreover, the production of a stable SC phenotype was caused by a loss-of-function mutation in some Solanaceae species (*Solanum pennellii, Solanum habrochaites*, and *Solanum arcanum*) (Kubo et al. 2015; Ma et al. 2021). Inactivation of *S-RNase* was observed in domesticated species with SC such as *S. lycopersicum,* while normal-functioning *S-RNase* was kept in wild populations showing SI (Encinas-Viso et al. 2020; Zhao et al. 2021b). Moreover, some Rosaceae species, except *Py. persica*, were shown to contain two or three copies of the *S*-like-locus, and these species regained SI probably due to inactivation or reduced expression of duplicated copies. The loss of SI in *Py. persica* may be due to the activation of both *S-RNase* and *S-like-RNase*, leading to the loss of SI by competitive interaction (Kubo et al. 2010; Zhao et al. 2021a). In addition to the three routes described above, a new route has recently been proposed to illustrate the loss of SI. In self-compatible diploid potato species, there is an *S*-locus inhibitor (Sli), which can interact with multiple allelic variants of pistil-specific *S-RNases*. The Sli was found to play a role as a general *S-RNase* inhibitor, converting self-incompatible potatoes to self-compatible potatoes (Ma et al. 2021).

### Origin and Specific Evolution of the *S*-locus in the Subfamily Maleae of Rosaceae

We investigated the evolutionary processes that the *S*-locus has undergone based on pairwise macro- and microsynteny analyses as well as *K*_a_/*K*_s_ calculation among Rosaceae fruit species with high-quality assembly. *S-RNases* emerged before the diversification of Rosaceae species, and subsequent gene loss of *S-RNases* occurred in Rosoideae species (e.g., strawberry Chr6) and Maleae species (e.g., pear Chr4 and Chr12). The ancestral *SLF* in the *S*-locus had an ancient origin, and the *S-RNase* of Rosaceae was originated more recent after the split of grape and Rosaceae. Rosoideae species inherited the *S*-locus from a Rosaceae ancestor, and subsequent loss of *S-RNases* occurred in strawberry and black raspberry. The ancestral *S*-locus containing *S-RNase* was also retained in Amygdaleae and Gillenieae species such as peach, Japanese apricot, apricot, and bowman’s root. Therefore, the *S*-loci in Rosoideae and Amygdaleae species had strong syntenic relationships. In Maleae, the *S*-*RNase* has undergone independent loss and gain events by using microsynteny analysis. *S-RNases* were lost from origin *S*-locus region on Chr 4 (12) which inferred would happen before a recent WGD, and independent origin of *S*-locus in the common ancestor of Maleae occurred after WGD, resulting in only one *S*-locus in Maleae species. Overall, the *S*-locus found in Maleae species has lost conserved synteny with that in Rosoideae and Amygdaleae species, which resulted from independent loss and gain events. Moreover, based on the calculation of *K*_s_ of *S-RNases* and *SLFs*, we found that *K*_s_ of “The ancestral *F-box*” on Chr 4 in apple and pear was similar to all *S-RNases* as well as “The ancestral *F-box*” gene in Amygdaleae and Rosoideae which linked to *S-RNase*s. Thus, it is inferred that *S-RNases* on Chr 17 in pear and apple (Chr 13 in loquat) of Maleae probably be derived from the translocation of the *S-RNase*s in primitive *S*-locus on Chr 12 or 4 (and in loquat, Chr 17 or 12) in the genome. In addition, we detected a number of *SLFs/S-like SLFs* in the *S*-locus of Maleae species, while only a few *SLFs* were found in Amygdaleae, indicating that *SLFs* in Maleae underwent additional single-gene duplication events during evolution, and the results of *K*_s_ showed that *SLFs* in Maleae species were generated more recently (supplementary tables S10 and S13, Supplementary Material online). The proliferation of *SLFs* may have led to the differences between the *S*-locus in Amygdaleae and Maleae species. However, whether or how these duplicated *SLFs* function in SI remains to be determined in future studies.

## Conclusion

In this study, we identified 785 RNase T2 genes in 81 sequenced plants covering diversified lineages. The RNase T2 genes were divided into three subgroups (Class I, II, and III). The Class I genes were found to have an ancient origin, while Class II (angiosperms-specific) and III genes (eudicots-specific) emerged more recently. Each of the three major classes could be further classified into several specific subclasses. Single-gene duplications and lineage-specific WGD contributed to the expansion of the RNase T2 family. The duplication, deletion, or inactivation of *S-/S-like-RNase* was inferred to be related to repeated loss and gain of SI in different plants. Moreover, the origin and evolution of *S*-locus in Rosaceae species were characterized by independent loss and gain of *S-RNase* in different lineages. This work lays a foundation for deeply understanding the evolution of the RNase T2 gene family and SI in plants.

## Materials and Methods

### Plant Genome Data Collection

In total, 81 plant genomes were included in this study (supplementary table S14, Supplementary Material online), including 60 eudicots, 11 monocots, basal angiosperm (*Amborella trichopoda*), gymnosperms (*Sequoiadendron giganteum* and *Ginkgo biloba*), fern (*Azolla filiculoides*), lycophytes (*S. moellendorffii*), bryophytes (*Ph. patens*), streptophyta (*M. viride*), chlorophyta (*C. reinhardtii*), and prasinodermophyta (*Pr. coloniale*). The genome data were downloaded from the comprehensive databases such as NCBI (Coordinators 2015), Phytozome (Goodstein et al. 2011), Genome Warehouse (Chen et al. 2021), and some specialized databases such as GDR (Jung et al. 2018), Sol Genomics Network (Fernandez-Pozo et al. 2014), and CuGenDB (Zheng et al. 2018). For each gene, the primary transcript and corresponding protein sequence were used.

### Pairwise Genome Comparisons and Syntenic Block Identification

Interspecies as well as intraspecies comparisons are required for using SynNet-Build pipeline to detect synteny blocks (Zhao and Schranz 2017). Thus, for 81 plant species investigated, we performed a pairwise whole-genome protein comparison between any two species and within species. Diamond which is a BLAST-like software but much more efficient was used with the default parameter. MCScanX was used to identify intra- and intergenomic collinearity using default parameters (minimum match size for a collinear block = 5 genes, max gaps allowed = 25 genes).

### Identification of RNase T2 Genes and Synteny Network Analysis

The seed alignment file of the RNase T2 domain (PF00445) obtained from the Pfam database (http://pfam.xfam.org/) was used for constructing an HMM file to identify candidate RNase T2 genes with *P*-value < 1e^−10^ using HMMER3 software (supplementary table S1, Supplementary Material online). The rows contained two RNase T2 genes were retrieved from the “Total Synteny Blocks” file and stored into a new file “Syntenic Blocks RNase T2 genes” (supplementary table S1, Supplementary Material online). This file incorporated the syntenic relationships among RNase T2 genes from 81 plants, and the network was visualized in Gephi 0.9.1. The script performing the “Pairwise Whole-Genome Comparisons” and “Syntenic Block Calculation” steps and more additional information about the SynNet-Build method can be found at GitHub (https://github.com/zhaotao1987/SynNet-Pipeline).

### Phylogenetic Analysis and Conserved Motif Identification

The species tree of 81 plants was constructed according to the taxonomy tree in NCBI Taxonomy Common Tree (https://www.ncbi.nlm.nih.gov/Taxonomy/CommonTree/wwwcmt.cgi) and refined according to some recent studies (Yang et al. 2015; Bombarely et al. 2016; Zhang et al. 2017, 2020; Chaw et al. 2019; Chen et al. 2019; Li et al. 2019; Yoshida et al. 2019). The full amino acid sequences of RNase T2 genes were aligned using MAFFT v7.475 (https://mafft.cbrc.jp/alignment/software/) with the parameters –genafpair –maxiterate. The multiple sequence alignment file was then used to construct RNase T2 phylogenetic tree using IQ-TREE (version 2.0.3) with the maximum-likelihood method. The bootstrap value was set to 1,000. The phylogenetic tree was visualized using iTOL v6. The Multiple Expectation–maximization for Motif Elicitation (MEME) tool was used to identify conserved motifs using parameters: maximum number of different motifs 20; minimum motif width 6, and maximum motif width 200. The results were visualized and appended to the phylogenetic tree using iTOL v6.

### Identification and Sequence Analysis of *S-RNases*

We obtained the reported *S-RNase* sequences from five plant families (Rutaceae, Rosaceae, Solanaceae, Plantaginaceae, Rubiaceae) with GSI from GenBank (https://www.ncbi.nlm.nih.gov/genbank/). These sequences were used as query to search against RNase T2 protein sequences in each species using Diamond, and the best hit gene was considered as the candidate *S-RNases*. Amino acid sequences of *S-RNases* were aligned using Clustal Omega (https://www.ebi.ac.uk/Tools/msa/clustalo/), and then ESPript 3.0 was used (https://espript.ibcp.fr/ESPript/ESPript/) to visualize the multiple sequence alignment results.

### Annotations of *S-locus* and *S-like*-locus *F-box* and *F-box-associated* Gene in *S*-locus and *S-like*-locus

We obtained *S-like-RNase* gene by performing BLASTP with Diamond against the protein databases of 81 species using amino sequences of the proteins encoded by well-known *S* genes as queries, BLAST was run using default setting with a cut-off of 10^−10^ (T2 RNases) and max-target-sequence was set to 5. In addition, the seed alignment file of the F-box domain (PF00646), F-box-associated (FBA) domain (PF07734, PF08268), and F-box-like domain (PF12937) obtained from the Pfam database was used for constructing an HMM file to identify candidate *F-box/FBA* genes with *P*-value < 1e^−10^ using HMMER3 software. Candidate genes in 1.5–3 Mb upstream and downstream regions of *S-RNase* and *S-like-RNases* were selected for detailed analysis. The genomic structures of GSI *S*-locus and *S*-like-locus were annotated based on the phylogenetic analyses and BLASTP results of Class III T2 RNases (supplementary fig. S11, Supplementary Material online) and their linked *SLFs/S-like SLFs*, some species were obtained some fragments, respectively, based on BLAST results, because the assembly of the genome sequences of these species is incomplete and still at the scaffold stage (*P. axillaris*, *C. medica*, *C. reticulata*; Liang et al. 2020).

### Macrosynteny and Microsynteny Analysis of *S*-locus Region

Seven Rosaceae species (*F. vesca*, *R. occidentalis*, *P. bretschneideri*, *M. domestica*, *E. japonica*, *Py. persica*, *Pr. mume*) were used in this section of analysis. The 1.5–3 Mb upstream and downstream region of *S-RNase* was extracted as the potential region for detailed analysis. Macrosynteny and microsynteny analyses focused on *S*-locus between two species and within same species were performed at chromosome- and gene-level using MCscan (Python version) incorporated in jcvi (https://github.com/tanghaibao/jcvi/wiki/MCscan-(Python-version).

### Duplicated Gene Pairs Identification and Nonsynonymous (*K*_a_) and Synonymous (*K*_s_) Calculation

Paralogous RNase T2 gene pairs derived from WGD, TD, PD, TRD, and DSD were identified using the DupGen_finder pipeline (https://github.com/qiao-xin/DupGen_finder). The nonsynonymous substitution rates (*K*_a_) and the synonymous substitution rates (*K*_s_) of syntenic gene pairs were calculated using the Nei–Gojobori method implemented in KaKs_Calculator 2.0 (Wang et al. 2010). Homologous gene pairs of *S-RNases* and *SLFs* used for *K*_a_/*K*_s_ calculation were obtained from BLASTP results.

## Supplementary Material

Supplementary data are available at *Genome Biology and Evolution* online.

## Supplementary Material

evac093_Supplementary_Data

## Data Availability

The data underlying this article are available in the article and in its online Supplementary material.
