# Supplementary material for: The Origin and Evolution of RNase T2 Family and Gametophytic Self-incompatibility System in Plants
Source: Genome Biol Evol. 2022 Jun 17;14(7):evac093. doi: 10.1093/gbe/evac093 (PMC9250077; doi:10.1093/gbe/evac093)
Supplement: evac093_Supplementary_Data [file evac093_supplementary_data.zip › Supplemental Figure.docx]

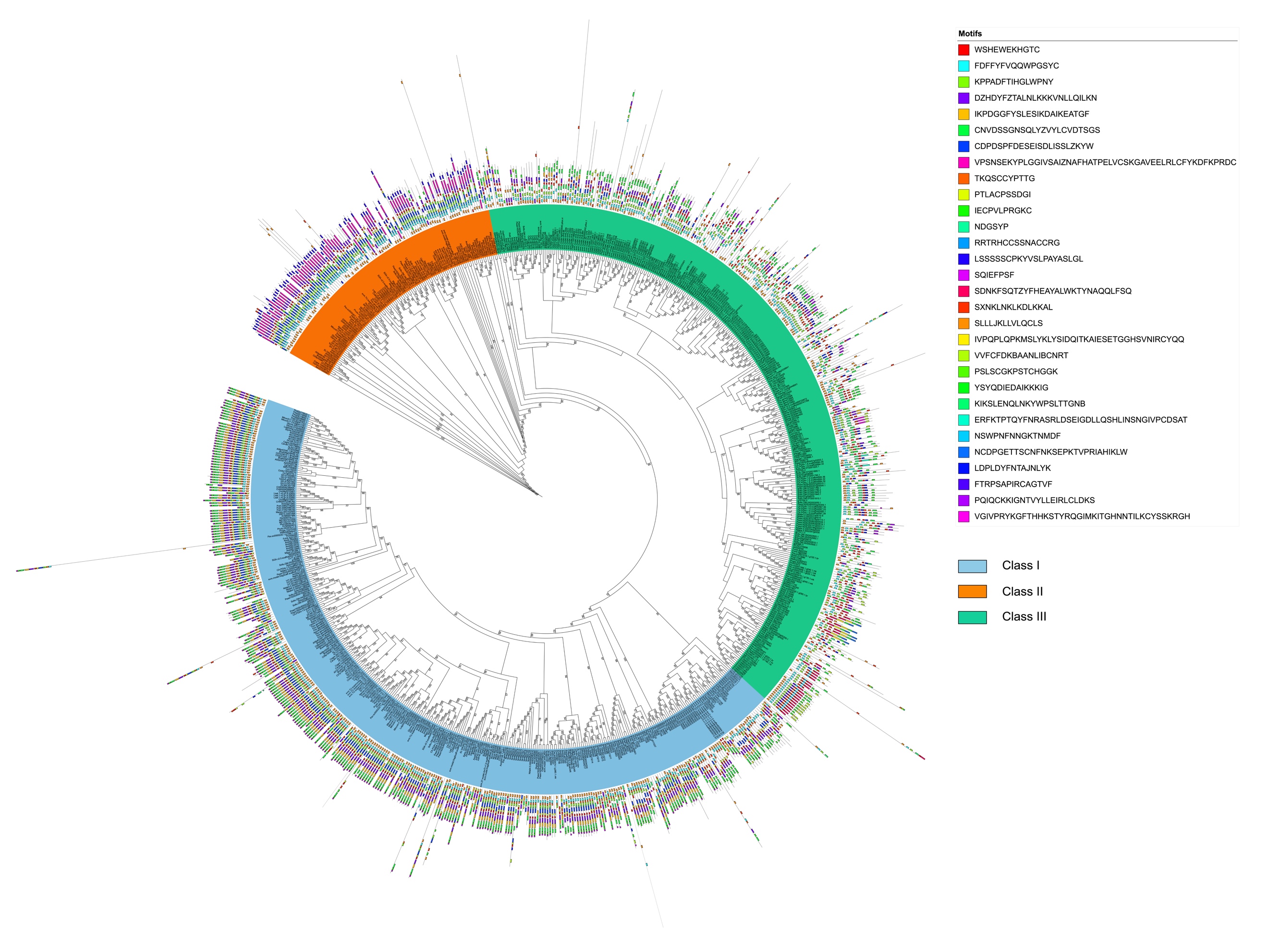


**Fig. S1. Phylogenetic trees of RNases T2 family genes in 81 plants.**

The phylogenetic tree of RNase T2 gene family with complete branch label was created by IQ-TREE using full-length protein sequences and visualized by iTOL v6.3. The bootstrap was set as 1000 replicates. Three different colors represent each Class, 20 motifs were label in each branch respectively.

**Fig. S2.** **Phylogeny of RNases T2 family genes in 81 plants**. The phylogenetic tree was constructed using IQ-TREE with the maximum-likelihood method and visualized using iTOL v6.3 with support values. The bootstrap was set to 1000 replicates. Tip labels have been omitted for clarity. The RNase T2 genes were divided into three major classes (Class I, II, and III). *S-RNase* genes within Class III are indicated in five families with GSI, including the Rubiaceae, Plantaginaceae, Solanaceae, Rutaceae, and two genera of Rosaceae (Amygdaleae and Maleae).

**Fig. S3. Phylogenetic trees of Class I members in RNases T2 family.**

The phylogenetic tree of Class I members in RNase T2 gene family with complete branch label was created by IQ-TREE using full-length protein sequences and visualized by iTOL v6.3. The bootstrap was set as 1000 replicates. Three different colors represent each Class, gene IDs were labeled in each branch respectively. Class I was divided into three subclasses.

**Fig. S4. Phylogenetic trees of Class II members in RNases T2 family.**

The phylogenetic tree of Class II members in RNase T2 gene family with complete branch label was created by IQ-TREE using full-length protein sequences and visualized by iTOL v6.3. The bootstrap was set as 1000 replicates. Three different colors represent each Class, gene IDs were labeled in each branch respectively. Class II was divided into three subclasses.

**Fig. S5. Phylogenetic trees of Class III members in RNases T2 family.**

The phylogenetic tree of Class III members in RNase T2 gene family with complete branch label was created by IQ-TREE using full-length protein sequences and visualized by iTOL v6.3. The bootstrap was set as 1000 replicates. Three different colors represent each Class, gene IDs were labeled in each branch respectively. Class I was divided into five subclasses.

**Fig. S6. Amino-acid sequence alignment of the *S-RNases* from 23 species.**

The conserved regions and the hypervariable regions of the *S-RNases* from Solanaceae and Rosaceae are indicated under the corresponding sequences. The *S-RNases* sequences of *Citrus sinensis* and *Citrus reticulata* are significantly shorter than others and lack conserved domain 5.


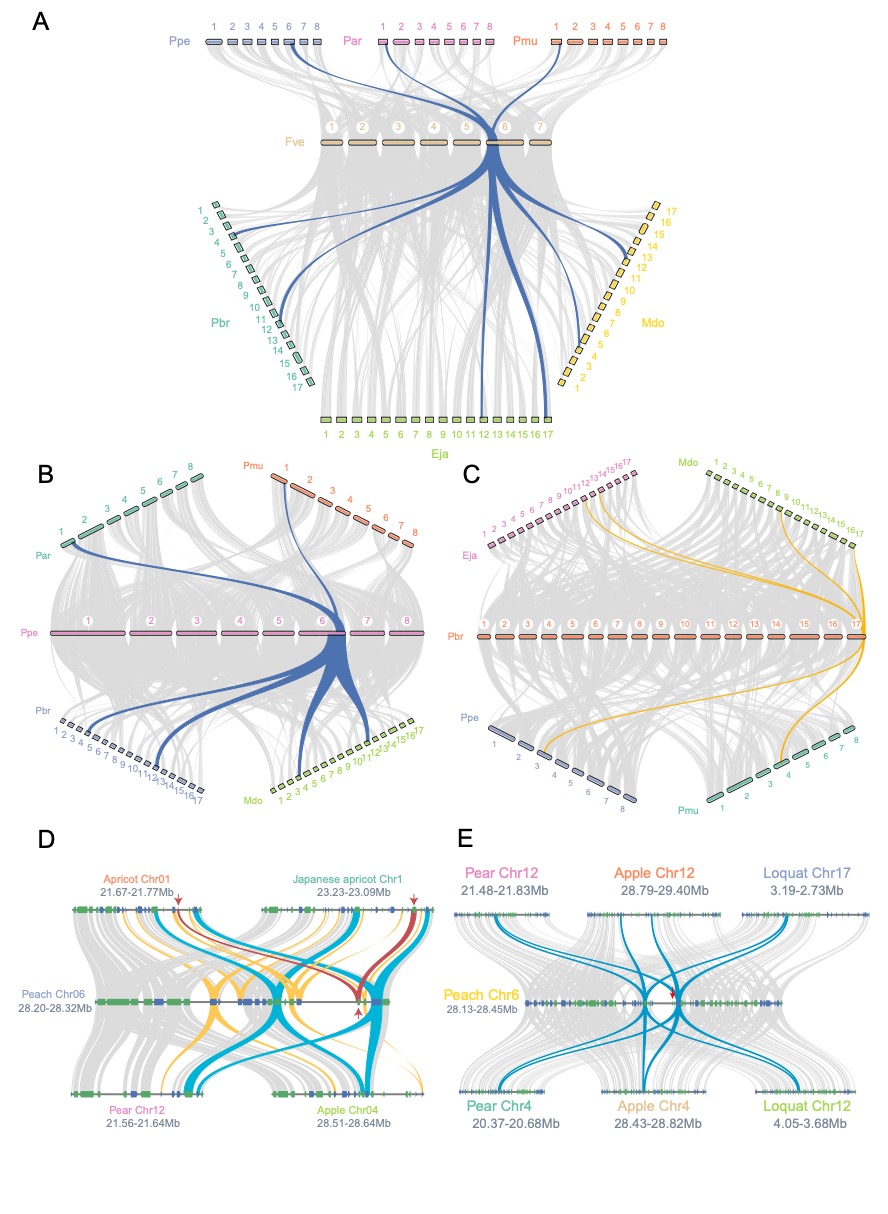


**Fig. S7. Macrosynteny and microsynteny analysis in Rosaceae. A.** Macrosynteny among seven species in Rosoideae, Amygdaleae and Maleae. Different colored blocks represent chromosomes in six species (Fve: *F. vesca,* Ppe: *P. persica,* Par: *P. armeniaca,* Pmu: *P. mume,* Pbr: *P. bretschneideri,* Eja: *E. japonica,* Mdo: *M. domestica*). Curves between blocks represent synteny relation. Synteny between *S*-locus in strawberry and its collinear regions in other species were colored by blue.

**B.** Macrosynteny among *S*-locus in peach and four other species. Different colored blocks represent chromosomes in five species. Macrosynteny based on peach shows that no detectable synteny between *S*-locus in Amygdaleae and *S*-locus in Maleae genomes. **C.** Macrosynteny among *S*-locus in pear and four other species. Synteny between *S*-locus in pear and its collinear regions were colored by yellow. Macrosynteny based on pear shows that no detectable synteny between *S*-locus in Maleae and *S*-locus in Amygdaleae genomes. **D.** Microsynteny relationships of *S*-locus region in peach with four other collinear regions among several species in Rosaceae. No synteny was found in *S*-locus regions in peach with its collinearity in pear and apple. Yellow curves represent *SFBs*, *S*-locus were flanked by the homologous genes connected by blue curves. **E.** Microsynteny relationships of *S*-locus region in peach with its collinear regions in pear, apple and loquat. Genes flanked on *S*-locus in peach have a strong synteny relation with genes in pear and apple, which means these segments shared a common ancestor. However, *S*-locus was not detected in pear/apple chromosome 4, 12 and loquat chromosome12,17, which means loss event happened in Maleae.

**Fig. S8. Phylogenetic trees of RNases T2 family genes in 81 plants.** Macrosynteny relationships in four species in Rosoideae, Amygdaleae and Maleae. Different blocks represent chromosomes. Curves between blocks represent synteny relation.


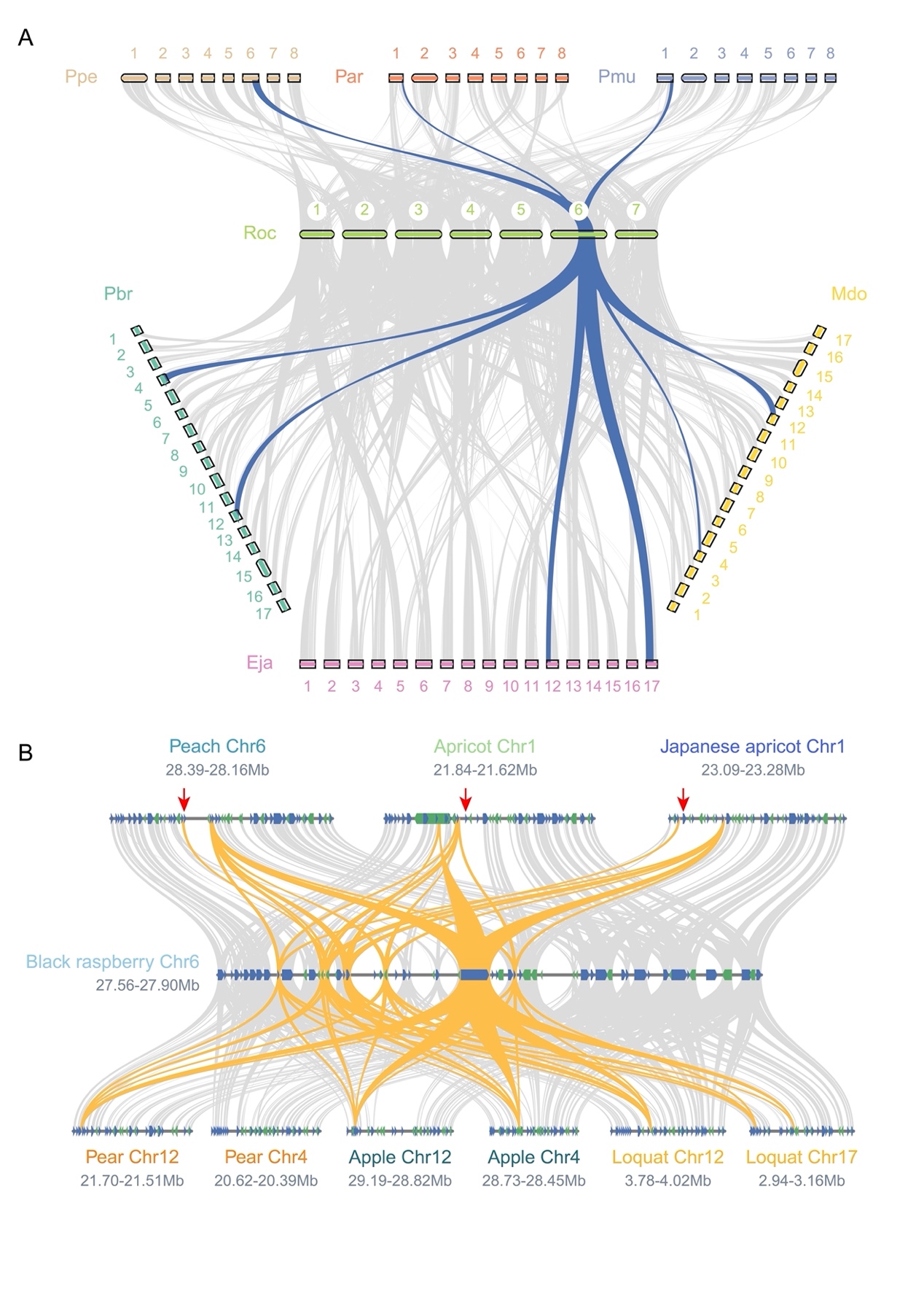


**Fig. S9. Macrosynteny and microsynteny analysis among black raspberry with other species in Rosaceae.** A. Macrosynteny among seven species in Rosoideae, Amygdaleae and Maleae. Different colored blocks represent chromosomes in six species. Curves between blocks represent synteny relation. Synteny between *S*-locus in black raspberry and its collinear regions in other species were colored by blue. B. Similar to results in strawberry, microsynteny can be observed among seven species syntenic regions, *S-RNases* were not detected in black raspberry Chr 6 and Maleae Chr 4 and 12. Red arrows are the location of S-RNases, yellow curves were the synteny relationships among *SLFs* genes in seven species.


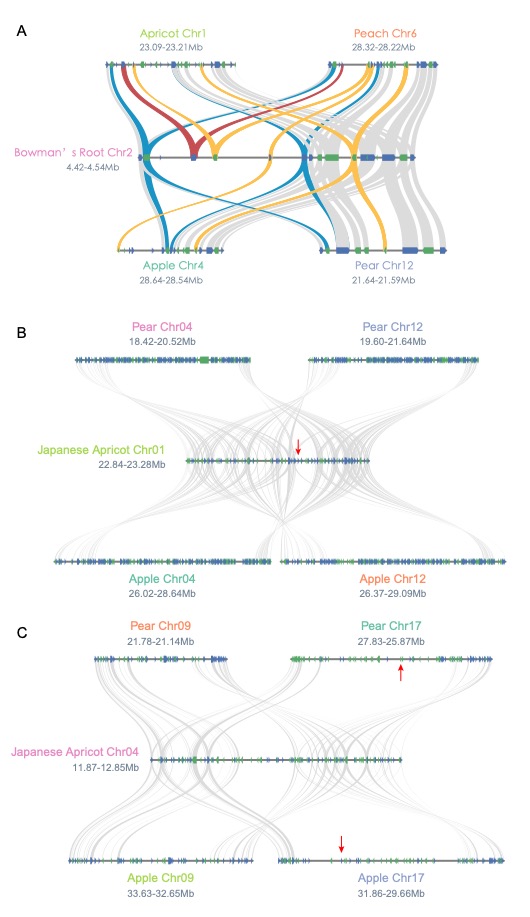


**Fig. S10. Microsynteny analysis among Japanese apricot with apple and pear in Rosaceae. A.** Microsynteny relationships of *S*-locus region in bowman’s root (*G. trifoliata*) with four other collinear regions among several species in Rosaceae. No synteny was found in S-locus regions in bowman’s root with its collinearity in pear and apple. Yellow curves represent *SFBs*, S-locus were flanked by the homologous genes connected by blue curves. B. Microsynteny relationships of *S*-locus region in Japanese apricot with collinear regions in apple and pear. No synteny was found in *S*-locus regions in Japanese apricot with its collinearity in pear and apple. Red arrows represent *S-RNases*. C. Microsynteny relationships of Chr 4 region in Japanese apricot with its collinear regions in. Genes flanked on Chr 4 in Japanese apricot have a strong synteny relation with genes in apple and pear, which means the segments on Japanese apricot Chr 4 and pear/apple chromosome 9, 17. Likewise, synteny relation between Japanese apricot Chr 4 and Chr 9 and Chr 17 in pear/apple suggested that *S*-locus in apple and pear only existed on Chr 17 while lost in Chr 9.

**Fig. S11. Phylogenetic trees of *S/S-like* genes in RNases T2 family members of 81 species.**

The phylogenetic tree of *S/S-like genes* in RNase T2 gene family with complete branch label was created by IQ-TREE using full-length protein sequences and visualized by iTOL v6.3. The bootstrap was set as 1000 replicates. Three different colors represent each Class.
